# Sleep well, worry less: A co-design study for the development of the SMILE app

**DOI:** 10.1177/20552076241283242

**Published:** 2024-09-25

**Authors:** Marcus Cormier, Matt Orr, Alanna Kaser, Hannah MacDonald, Jill Chorney, Sandra Meier

**Affiliations:** 1 Department of Psychiatry, 3688 Dalhousie University, Halifax, Canada; 2 Department of Psychology, 8689 Acadia University, Wolfville, Canada; 3 Department of Public Health Science, School of Medicine, 4257 Queen's University, Kingston, Canada; 4 545971 Mental Health and Addictions Program, IWK Health, Halifax, Canada

**Keywords:** Anxiety, sleep problems, youths, mobile apps, mobile sensing, eHealth

## Abstract

**Objective:**

With the coronavirus disease 2019 pandemic exacerbating mental health concerns, the prevalence rates of anxiety and sleep problems have increased alarmingly among youth. Although 90% of patients with anxiety experience sleep problems, current interventions for anxiety often do not target sleep problems in youth. Given this lack, we designed the SMILE app, an intervention that addresses both anxiety and sleep problems simultaneously.

**Methods:**

As users’ perspectives are essential to ensure app engagement and uptake, the features, designs, and functions of the SMILE app were evaluated using a participatory app design approach. Participants (*N *= 17) were youth aged 15 to 25 who reported co-morbid anxiety and sleep issues above clinical thresholds. After completing an online screening survey assessing demographics, anxiety, and sleep problems, participants shared app feedback through group-based, semi-structured co-design sessions. Qualitative analyses were conducted to identify common themes from participants’ feedback.

**Results:**

While participants expressed enthusiasm for the SMILE app's features, particularly the *Visualization, Journaling, and Psychoeducation* features, and their variety, they criticized the design aspects of the app, such as the font and text amount. Most participants stated they would use the SMILE app or recommend it to a friend.

**Conclusion:**

By actively involving the target population in the design process, the SMILE app has the potential to notably improve the mental well-being of youth, though further research and development are required to realize this potential fully.

## Introduction

Anxiety disorders are among the most common mental health problems among youth, with an estimated 6-month prevalence of 11–15%.^1^ Additionally, the coronavirus disease 2019 pandemic has further exacerbated the prevalence of anxiety problems among youth.^2,3^ Anxiety problems in youth are associated with a variety of negative outcomes (e.g. alcohol abuse, suicide, academic failure).^4–6^ Many youths with anxiety problems also experience additional health problems, for example up to 90% of these youth report sleep problems,^1^ which can aggravate their anxiety.^7^ The relationship between anxiety and sleep problems is theorized to be bidirectional. Sleep problems may contribute to the development of anxiety problems, which in turn may be antecedents of sleep problems.^8^ Previous empirical studies have supported both pathways. De Bruin et al.^9^ found that anxiety problems in youth are associated with significantly longer sleep onset latency and significantly shorter sleep duration. Despite the high overlap and interrelatedness of anxiety and sleep problems in youth, standard interventions for anxiety do typically not target sleep problems.^8^

Moreover, many youths with sleep problems face barriers to receiving services.^10^ Yet, limited access to mental health services and long wait times can profoundly impact youth by decreasing treatment motivation, prolonging physical and emotional distress, and exacerbating mental health conditions.^11,12^ Mental health apps have been suggested as a possible solution for addressing unmet needs for youth mental health support.^11,13^ Many youths even prefer app-based solutions^9^ over traditional services as mental health apps are easily accessible, anonymous, and can monitor mental health symptoms in real-time to provide immediate support.^14^ Recent reviews demonstrated that mental health apps can effectively reduce anxiety in youths, but the reviewed apps rarely target sleep problems.^15^ Some available mental health apps address both anxiety and sleep problems. However, these apps are primarily designed for, and used by adults, and are often static in design (i.e. do not change based on user input).^16^

The few youths who use these adult-targeted apps find them difficult to navigate and commonly expressed dislike for the lack of personalization.^16^ Personalizing mental health apps to increase youth user engagement can be challenging. New advances in technology, such as mobile sensing (i.e. the collection of behavioral data through mobile phones),^17^ may help advance the personalization of interventions to the individual needs of users. Specifically, mobile sensing offers unprecedented opportunities to identify and understand distinct areas where youth experience problems by passively recording their daily life behaviors.^18^ Using mobile sensing data, intervention features displayed to youth users could be highly tailored to their problem areas. Thus, mobile sensing offers promising avenue to address the lack of targeted and personalized e-mental health interventions for youth experiencing anxiety and sleep difficulties.

Sleep tracking has been shown to be a highly effective component of sleep interventions.^19^ Sleep can be successfully measured via mobile sensing. Specifically, mobile-sensed sleep measures have recently been shown to correlate well with actigraphy data and sleep diaries.^20^ Mobile sensing can also help uncover maladaptive behaviors that could contribute to anxiety and sleep problems. For example, mobile sensing data can help identify avoidance patterns that may hinder youth in overcoming their anxiety problems (e.g. lack of social contacts via call and text message logs),^21^ spending time primarily at home via GPS data.^17,22^ Logging screen activity, noises, and light mobile sensing can also uncover bad sleep habits, such as high screen time before bed or a very loud environment.^23^ Most intervention apps rely on self-reports for behavior tracking.^24^ However, the completion of daily ratings may be too burdensome, especially for youths.^25,26^ Passive tracking could reduce the perceived usage burden and potentially help mitigate app attrition.^25–28^ While mobile sensing may raise certain privacy concerns, a recent survey outlined the acceptability and usability of mobile sensing by youth patients and their parents to increase awareness of poor habits and its potential to promote behavioral change.^28^ In line with these concepts, the Smile for Life (SMILE) app was developed, leveraging mobile sensing to measure maladaptive behavior and personalize intervention content for youth with anxiety and sleep problems.

To ensure that the newly designed SMILE app could truly address the needs of youth with anxiety and sleep problems, a co-design study involving 17 youth was conducted. This allowed youth with sleep and anxiety problems to see and evaluate the app and ultimately influence the app's design and features. Evaluating the SMILE app with youth users was imperative, as research has shown that independent of their evidence-based content, mental health apps are often not well adopted. Approximately 70% of patients who used or were invited to use mental health apps stopped prematurely or declined, with lack of engagement cited as a major contributor.^29^ The objectives of the study thus were to examine youth users’ perspectives to identify facilitators and barriers of use and to reveal specific features, designs, and functions that may help with engagement and uptake of the SMILE app.

## Method

This qualitative co-design study was approved by the IWK Health Centre research ethics committee (approval no. 1026748) on 11 May 2021.

### Participants

Study participants included youths between the ages of 15 and 25 who self-identified as experiencing co-morbid anxiety and sleep problems and were able to speak and read English fluently. Participants were excluded if they did not own a smartphone. Youth were recruited using two approaches. First, advertisements were posted at local clinics in Halifax, NS, Canada, where the study took place. Second, advertisements were posted on various social media platforms (e.g. Instagram, Facebook, Twitter). Recruitment was conducted between November 2022 and April 2023. Seventeen youth attended co-design sessions. Participants who attended co-design sessions were compensated for their time with a $20 Amazon gift card.

### Procedure

The screening, consent, and online survey were completed online via the secure Research Electronic Data Capture (REDCap)^30^ platform. Interested youth who clicked on the hyperlink embedded in advertisements were automatically directed to an eligibility screener. The screener included seven items, which asked participants their age and six yes or no items that assured participants (1) experienced anxiety, (2) experienced sleep problems, (3) could read English, (4) could speak English, (5) currently owned a smartphone, and (6) were interested attending a co-design session. Interested participants who met inclusion criteria were then guided through a detailed study information and consent process, which was necessary to participate. Once participants provided their written (electronic) informed consent, they reported their demographic information, anxiety, and sleep problems in the form of a short survey. After survey completion, participants were invited via email to participate in one-on-one or group-based co-design sessions. The co-design sessions were conducted in person at the IWK Health Centre and via Zoom by two of the authors (M.O. and A.K.). The sessions lasted approximately 60 min and were carried out using a semi-structured guide. The guide facilitated author' questions and included demonstrations of SMILE app prototypes to obtain relevant user feedback on the SMILE app and its features, as well as participants’ general opinions on mental health apps and their features (see Supplemental Material S1). During sessions, participants also had opportunities to interact with SMILE app prototypes. All sessions were audio recorded and transcribed verbatim. Changes or recommendations to the sleep intervention features were prioritized for future development if it was mentioned by at least 10% of the sample during sessions.

### Measures

#### Demographic questionnaire

The demographic questionnaire asked participants to report their age, gender, sex, ethnic or cultural heritage, and community (i.e. rural, town, city under/over 500,000).

#### Screen for child anxiety related disorders

To measure anxiety problems, participants who reported that their age was between 15 and 18 completed the validated 41-item Screen for Child Anxiety Related Disorders (SCARED). Participants responded to questions about their anxiety symptoms/experiences as not/hardly ever true (0), somewhat/sometimes true (1), or very/often true (2). The SCARED demonstrates good internal consistency (α = 0.86–0.97) and good discriminant validity between anxiety and other disorders and within anxiety disorders for generalized and social anxiety.^31^ All youth participants endorsed scores greater than or equal to 25 indicating the presence of an anxiety disorder.

#### Screen for adult anxiety related disorders

To characterize the anxiety problems, participants who reported their age was between 19 and 25 completed the validated 44-item Screen for Adult Anxiety Related Disorders (SCAARED). Participants responded to questions about their anxiety symptoms/experiences as not/hardly ever true (0), somewhat/sometimes true (1), or very/often true (2). The SCAARED demonstrates good internal consistency (α = 0.90) and good discriminant validity between anxiety and other disorders and within anxiety disorders for generalized and social anxiety.^32^ All adult participants endorsed scores greater than or equal to 25 indicating the presence of an anxiety disorder.

#### Insomnia severity index

To obtain information on sleep problems, all participants were asked to complete the validated seven-item Insomnia Severity Index (ISI). Participants were asked to report the severity of their difficulty falling asleep, staying asleep, and waking up too early on a 4-point scale (i.e. 0 = none, 4 = very severe). They also were asked to report how satisfied they are with their sleep, how noticeable they believe their sleep problems are, how worried/distressed they are about their sleep problems and the extent to which their sleep problems interfere with their daily functioning. The ISI demonstrates good internal consistency (α = 0.90 and 0.91) and good discriminant validity between symptoms of insomnia.^33^ Scores between 8 and 12 indicate subthreshold insomnia, scores between 15 and 21 indicate moderate clinical insomnia and scores between 22 and 28 indicate severe clinical insomnia. All participants endorsed at least subthreshold insomnia levels (*n *= 4), with a majority endorsing clinical insomnia (*n *= 13).

#### Co-design interview guide

The author-created interview guide (see Supplemental Material S1) was adapted from the framework of Werner-Seidler et al.^34^ in consultation with youth, service providers, and clinician-scientists. The interview guide asked participants about their use of mobile apps, and their opinions of the SMILE app (e.g. which features they liked or disliked, what they would improve, if they would recommend it). The same interview guide was used during all group interviews. Co-design session interviews were conducted by M.O. (a male post-doctoral fellow, PhD) and A.K. (a female undergraduate research assistant, B.Sc.). Interviewers expanded beyond the interview guide (i.e. probes and prompts) throughout each interview to promote elaboration on given responses. To ensure that input was received from all participants, interviewers sought to encourage responses through direct questioning, provision of elongated pauses, and reflective listening.

### SMILE app

**Figure 1.**
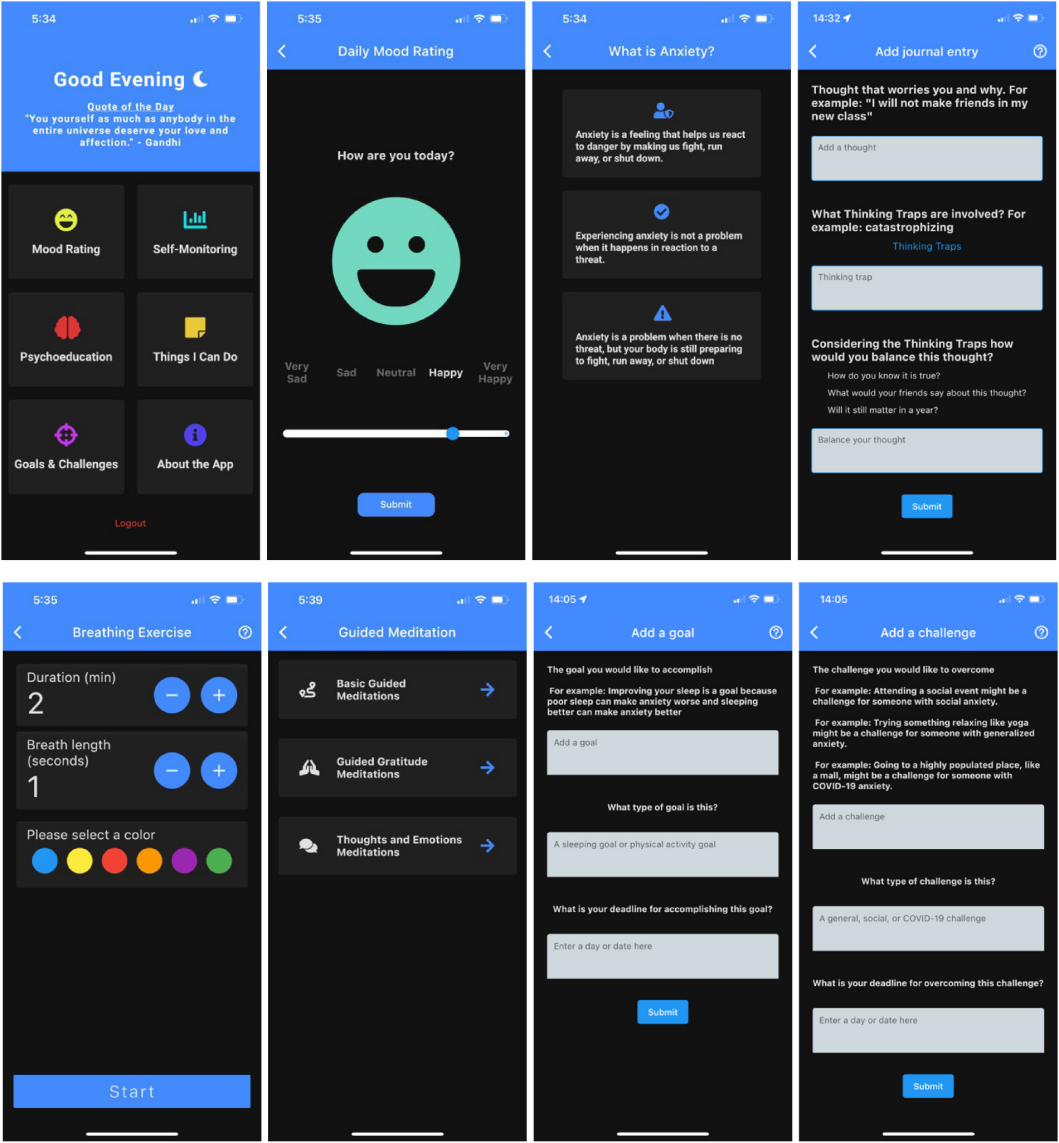
SMILE app.

The SMILE app (see Figure 1) was developed from a cumulative research and parallel consultation approach. Consultation with youth consultants, service providers, and clinician-scientists informed the initial prototype app development. App features were consequently developed to target anxiety and sleep problems via evidence-based techniques in line with the suggestions of the consultants. For example, the *Psychoeducation* feature was added to help youth learn about the different facets of anxiety problems.^35^ The *Journal* feature was included considering the long-term benefits evidenced by expressive writing.^36^ Based on the wishes of youth consultants, the *Journal* feature enables searches of previous entries and supports speech-to-text functions. The *Relaxation* and *Guided Meditation* features support self-regulation and decreased physiological arousal,^37^ and diverse types of mediations presented in different modalities were included as suggested by the consultants. The *Mood Tracking* feature was designed to encourage attention toward positive (versus negative) moods and help users understand and manage their emotions.^38^ The *Visualization* feature was included to increase self-awareness of daily behaviors^39^ (i.e. sleep, physical activity, and screen time), as many consultants expressed an interest in simple visualizations. The *Challenges* feature was included to motivate exposure to feared stimuli so that users learn and understand that their anticipated negative consequences are ill-founded.^40^ Service providers and clinician-scientists not only highlighted the value of the *Challenges* feature but also remarked on the need to ensure that the selected *Challenges* are not too demanding for users. Accordingly, the *Challenges* features only present tasks of moderate complexity and intensity. The *Goals* feature was designed to stipulate behavioral activation.^41,42^ The final version of SMILE app is currently planned to be accessed by users as a stand-alone University Health Services resource.

### Data analysis

The analysis consisted of a mixture of quantitative and qualitative methods and took place between April 2023 and October 2023. Descriptive statistics were used to characterize the sample in terms of demographic characteristics, anxiety, and sleep problems. M.O., M.C., and H.M. were the only research team members involved in data analysis. M.O. checked transcripts for accuracy before data analysis and imported them into NVivo Software 12. Inductive content analysis was used to analyze the transcripts of the co-design sessions.^43^ Content analysis was guided by the descriptive methods suggested by Braun and Clarke^44^ and Hsieh and Shannon.^45^ Each coder familiarized themselves with the data (i.e. read through all transcripts), and then each coder generated initial codes. Each coder then re-read the transcripts and coded instances of each initial code. These steps were all done independently. Coders M.C. and H.M. each independently coded half of the transcripts for reason of availability, while coder M.O. independently coded all transcripts. All coders then met to review and revise codes. Finally, codes were defined and categorized by consensus among coders. Using the criterion suggested by previous studies of eHealth interventions, which have used participatory design, a change or recommendation to the intervention features will be prioritized for future development if it is mentioned by at least 10% (i.e. 2) of the sample.^46^ This low threshold for prioritization was also used to ensure minority voices were heard and prioritized. M.O., M.C., and H.M. performed the coding, categorization, and mapping, and discussed all steps with the last author (S.M.). Discrepancies throughout the analysis process were resolved by consensus. Data saturation was based on category saturation and the richness (quality) and thickness (quantity) of the data.^47^ While data saturation was achieved with 12 participants (i.e. interviews and analytic procedures provided no new material for analysis), five additional youth participated in co-design sessions given the interest of youth in the study.

## Results

### Participants

Of the 117 participants who were screened for eligibility, most (*n *= 70, 60%) were not eligible (*n *= 4 were too young, *n *= 11 were too old, *n *= 13 reported no anxiety problems, *n *= 8 reported no sleep problems, and *n *= 2 were not interested in participating in a co-design session) or did not complete informed consent (*n *= 32). Of the remaining 47 participants who completed consent, most (*n *= 30, 64%) dropped out of the study before completing participation due to scheduling difficulties. The remaining 17 participants fully completed the study. Participants who completed the study did not differ in their demographics or clinical symptoms from participants who could not attend the co-design session due to scheduling conflicts.

### Sample characteristics

Table 1 presents the demographic characteristics of the 17 participants who completed the co-design sessions. The average age was 20.29 years (*SD *= 2.59). Most of the participants reported their gender (*n *= 13, 80%) and sex (*n *= 14, 82%) as female, identified themselves as White (*n* = 8, 47%), and were living in a city with a population less than 500,000 (*n* = 9, 53%). For participants who completed the SCARED (*n *= 2), the average score was 57.50 (*SD *= 12.02). For those who completed the SCAARED (*n *= 15), the average score was 55.73 (*SD *= 15.05). These average scores are above the clinical thresholds for anxiety disorders (i.e. greater than or equal to 25). The average score on the ISI was 22.12 (*SD *= 4.74), indicating an average level of sleep problems that are equivalent to severe clinical insomnia.

**Table 1.** Demographic characteristics of participants.

Characteristic	Sub-categories	Values
Age		*M *= 20.29, *SD *= 2.59
Gender		
	Male	*n *= 3 (18%)
	Female	*n *= 13 (76%)
	Non-binary	*n *= 1 (6%)
Sex		
	Male	*n *= 3 (18%)
	Female	*n *= 14 (82%)
Cultural heritage		
	White	*n *= 8 (47%)
	Black	*n *= 1 (6%)
	South Asian	*n *= 5 (29%)
	Chinese	*n *= 1 (6%)
	Arab	*n *= 1 (6%)
	Southeast Asian	*n *= 1 (6%)
Community		
	Rural	*n *= 1 (6%)
	Town	*n *= 5 (29%)
	City under 500,000	*n *= 9 (53%)
	City over 500,000	*n *= 2 (12%)

Note: Mean (M) and standard deviation (SD).

### Qualitative feedback

Table 2 presents the feedback participants provided on the SMILE app and its features. We identified the five most frequent categories of responses from participants including *Likes*, *Dislikes*, *Concerns*, *Suggestions*, and *Potential Uptake*. Each of these categories included many sub-categories and codes within them; however, codes were only included if mentioned by at least two participants. The number of unique mentions of a code by all participants was tallied.

**Table 2.** Content analysis results.

Category of response	Example from text	Number of mentions
**Likes**		**248**
*App features*		121
Relaxation	I personally use that because my mind wanders a lot and I have severe anxiety issues, so breathing techniques help me calm down	20
Visualization	I love seeing that data visually	31
Fun and play	I really liked mostly the visual parts of the psychoeducation section, because … I feel like it kind of gives the app a little bit of personality, almost makes it kind of seem like a game	6
Goals and challenges	I feel like I would be more willing if I could see like, OK, in a week I’ll have this as an end goal rather than the arbitrary like ‘going to bed early’ type of thing	3
Journaling	I liked that you can write down your thoughts and rate your mood	18
Structured strategies	I think you know the sleep and the physical activity recommendations are, you know, really great. Obviously, they are going to be different depending on the person	17
Variety	I like the things I can do, I think there's something that not every app has	24
*Accessibility*		45
Easy to use	I actually really love the layout and feel it makes regular tracking easy	10
Low commitment	One of the things I like about it is that you don’t have to answer the questions if you don’t want to, you can just skip right past them.	9
Manageable goals	Like kind of like small steps of like we’ll start slow and then work up to kind of that bigger goal.	12
Psychoeducation	Yeah, that appeals to me. Just having like, tips and tricks and, like, the educative aspect would be nice too	16
*App design*	The colors and everything are appealing	25
*Autonomy*	What appeals to me is basically it will let you, like, it will give you suggestions based on your pattern… on what should I do to improve my sleep	7
*Customizability*	I liked that there was the option for the like, shorter little meditations, like even just like 5–10 min	17
**Dislikes**		**70**
Design	it's just overly crowded	22
Goals and challenges	I kind of internalize my own goals and challenges in life in general	3
Mood tracking	If somebody's asking me, like, ‘how do you feel?’, I like, I have no idea	7
Journaling	to me, that would just make me think about it more	6
High commitment	daily logging, because I would forget to do that	11
**Concerns**		**25**
Accuracy	I think would be best well paired with some sort of [other] device because I guess the screen time thing …might not be as reliable	11
Privacy	I’m very conscious when it comes to tracking and if an app like this asked me to track, I don’t know honestly if I would	7
**Suggestions**		**96**
*Accessibility*		32
Accessible design	I think the text is pretty small and maybe the font colour isn’t very contrasted with the image so that is reducing the readability	7
Notifications	A simple notification kind of thing, just like to remind you to open this app every day	17
Variety in suggestions	If it told me to do different things every day, I think I would be more likely to do that than if it just gave me the same tips every day	4
*Features*		23
Sleep psychoeducation	information about what the different sleep disorders or like, what the different sleep issues might be	5
Tracking	you could see your weekly average and your past weekly averages… versus like the last like 60 days versus the last year or whatever cause it can let you see kind of like the progression and if you’re on like an upward or downward trend	5
Interface		38
Aesthetic	I think maybe if you guys had like a feature where you kind of change the colour scheme yourself	26
Widget	you know how, like on some phones you can have like widgets and stuff?	5
**Potential uptake**		**34**
Would not use	I personally have not seen anything substantially different from what I would have my two help apps do	2
Would use	As soon as this app is available, I will be downloading it… I have a lot of friends who I think would benefit from this	20
Likely to recommend	Yeah, absolutely… I think a lot of different family and friends would probably be able to use different aspects of the app	12

***Likes****.* Responses were coded as likes when participants indicated they positively viewed any aspect or feature of the app. Participants consistently reported that they liked the *App Features* (*n* = 121). Participants particularly highlighted the appeal of the *Visualization* (*n *= 31) feature, which they considered to be simple, practical, and aesthetically pleasing. For example, one participant said, “I love seeing that data visually,” and another said, “I like the design of it, it is pretty.” Notably, participants strongly enjoyed the variety of features included in the app (*n *= 24). Participants indicated that this app had more features in one place compared to other health apps. Other participants enjoyed the *Accessibility* of the app (*n *= 45). They felt the app was easy to use (*n *= 10), with one participant explaining “I actually really love the layout and feel it makes regular tracking easy.” Participants also enjoyed the provision and readability of the *Psychoeducation* feature in the app (*n *= 16). Moreover, although the *Design* of the app overall received mixed reviews, participants did highlight that the app was simple, calm, and palatable.

***Dislikes.*** Responses were categorized as dislikes when participants indicated they negatively viewed any possible aspect or feature of the app. Most commonly, participants disliked the *Design* of the app (*n* = 22). Specifically, participants noted that they did not like the fonts or amount of text used in the app. In addition, some participants (*n *= 11) worried that using the app would require *High Commitment*, and that this would make the app unapproachable for casual use. One participant said when discussing what they did not like said, “daily logging, because I would forget to do that.”

***Concerns.*** Responses were coded as *Concerns* when participants indicated fear, uncertainty, or doubt about an aspect or feature of the SMILE app. The most common concern from participants was about the app's *Accuracy* (*n = *11). Participants indicated they were concerned that the app's screen-time-based mobile sensing would not be sophisticated enough to track their sleep at night, such as knowing mid-night wakeups and sleep quality. For example, “I think [this app] would be best well paired with some sort of [other] device because I guess the screen time thing …might not be as reliable.” *Privacy* was mentioned seven times by the participants as a concern they had about the app. Participants let us know that they feared that their personal data was potentially at risk due to the app's mobile sensing.

***Suggestions.*** Responses were coded as *Suggestions* when participants indicated aspects or features which they would add, change, or take away from the SMILE app. Participants most often suggested a customizable aesthetic (*n* = 26) and daily push notifications (*n* = 17). Participants indicated that the font and color choices could be difficult to read by color-blind or visually impaired users. One participant said, “I think the text is pretty small and maybe the font colour isn’t very contrasted with the image so that is reducing the readability.” Participants thought that a daily reminder from their phone to use the app would help them remember to use the app and stay on track with the tips provided. Furthermore, a few participants suggested that the feedback and health tips the app provides could include more variety. Participants let us know that receiving the same feedback from the app multiple times over would be discouraging and uninteresting to continue to try.

***Potential Uptake.*** Lastly, responses were coded as *Potential Uptake* of the SMILE app when participants indicated whether they would use or recommend the SMILE app. The majority of participants (n = 15) would use and recommend the app.

## Discussion

The escalating rates of mental health problems among youth, compounded by the limited accessibility of mental health services,^48^ necessitate innovative solutions, with mental health apps emerging as a promising avenue. Emphasizing a comprehensive approach, the National Canadian eHealth guidelines advocate for a multi-method strategy in the development and evaluation of mental health apps, explicitly including the perspectives of youth users to ensure acceptance.^13,49^ Recognizing the significance of these perspectives in tailoring apps to individual needs and stipulating the acceptance and uptake of mental health apps, the objective of the current study was to collaborate with youths in the design of a novel mental health app leveraging mobile sensing to improve anxiety and sleep problems.

Overall, the results of the study affirm the acceptance of the SMILE app among youth with anxiety and sleep problems, as evidenced by the high number of positive comments (*Likes*, n = 248). The app's perceived ease of use, accessibility, and integration, attributed to its diverse features, resonated well with the youth. Their endorsement of a wide range of features underscores the importance of variety in enhancing the acceptance and utilization of mental health apps, aligning with existing research.^50,51^ Additionally, the expressed willingness of most youths to use or recommend the SMILE app to a friend or family member with mental health problems indicates positive attitudes, a crucial factor in predicting acceptance and long-term engagement with mental health apps.^52,53^ Amid these generally positive perspectives, the invaluable feedback provided by youth serves as a foundation for future improvements to the app, ensuring alignment with their needs and promoting sustained acceptance and use.

Addressing a common barrier to user engagement, the SMILE app endeavors to overcome issues related to personalization by tailoring intervention features to the mobile-sensed daily behaviors of youth users,^54^ aligning with the broader paradigm shift towards precision medicine in mental healthcare. The appeal of the app's visualization features, which present mobile-sensed behaviors through simple graphs, highlights the potential of leveraging mobile sensing for effective self-monitoring and mental health care.^55^ Importantly, features, such as mood tracking, activity recognition, and sleep pattern analysis, have contributed to a more comprehensive understanding of users’ mental health, fostering a sense of agency and self-awareness.^56^ Self-monitoring and the ability to identify patterns in emotional responses and behaviors are further integral to many forms of psychotherapy.^57^ Consistent with our findings, self-monitoring seems to be a widely valued and desired aspect by mental health app users.^51,58–60^

While youth appreciated the enhanced tracking and self-monitoring features of the SMILE app, concerns regarding the accuracy and privacy of mobile sensing data also emerged. Mobile sensing has enormous potential to enhance healthcare, but it is not without risks.^61,62^ This is a perspective that many youth participants seemed to share. Privacy concerns must be taken seriously and addressed by clear communication. Previous research suggested that perceived control and trust can influence the privacy protection behaviors of users, and that trust is enhanced by offering individual choices.^63^ Future iterations of the SMILE app should incorporate specific permissions for mobile sensing features to enable users to choose what behaviors will be tracked. Similarly, the app should provide even clearer information on how mobile sensing contributes to the personalization of the app and emphasize the state-of-the-art techniques and safeguards in place to protect user privacy.

The design of the SMILE app emerged as a focal point for improvement. Youth provided both positive and critical reviews of the app's design but were also eager to provide design recommendations. Some common additions, from the many, suggested, included the ability to customize app visuals and text (i.e. color scheme, font style/size) and add a built-in notification system or a widget to increase personalized usage and engagement. Aligning with prior research on mental health apps, users desire a diverse array of features that are flexible or customizable enough to accommodate various needs.^51,58,64,65^ Theoretically, by supporting user-customizable features, messages within the app can be tailored to users’ needs and interests, which can then have greater potential for deep (rather than shallow) processing; this is in line with the Elaboration Likelihood Model of Persuasion^66^ and the Persuasive Systems Design Model.^67^ Enhancements of existing features were also recommended to boost utility and ease of use. Youth suggested extending the app's tracking abilities by requesting access to older data and data visualizations of behavioral changes across longer time frames in the *Visualization* features. Concurrently, prior research highlights enhanced tracking ability as a common user-requested app feature.^58,68^ More information on sleep problems in the *Psychoeducation* feature was another enhancement request. Positive user experiences of youth are often associated with engaging, interactive, and personalized psychoeducational content, further emphasizing the importance of tailoring information to individual needs.^69^

Incorporating user feedback plays a pivotal role in ensuring functionality and continued use of mental health apps.^50,51^ Users are unlikely to persist with apps that fail to engage them from the start,^70^ and neglecting user feedback can impede vital technical improvements, setting back evaluative research and the overall clinical effectiveness of an app.^29,70^ Consequently, the developers of the SMILE app emphasize a commitment to considering youth user perspectives and recommendations at each stage of development and implementation to ensure the app remains user-focused, engaging, and effective.

### Limitations and future directions

It is essential to acknowledge the limitations of the study and delineate potential avenues for future research. While the study collected a diverse range of perspectives from youth with anxiety and sleep problems, the results represent participants’ opinions and may not capture the views of all youth facing these issues. Moreover, caution due to sampling bias is recommended, as a sizable number of participants did not attend the focus groups despite some confirming their interest and availability. Those without the resources and/or motivation to attend the session may hold different opinions from those who attended. Assessing app acceptance among youth who declined participation may have resulted in more negative feedback. Additionally, participants may have been biased to respond positively due to sharing feedback directly with researchers during sessions. Negative feedback is essential for informing the continued adaptation of the app to meet the diverse needs of youth.^29,71^ Future research should consider strategies to seek critical feedback, mitigate bias, and ensure youth perspectives are representative. Despite efforts to recruit an ethnically diverse sample, the qualitative focus and small sample size limit representation across certain demographic groups and broad generalizability. The predominant representation of females among the participants calls attention to the well-documented sex and gender differences in the acceptance of new technology^72,73^ and outlines the need for future research that includes more diverse gender identities. In addition, more information is required on the socioeconomic background of users in future research, as prior research has established links between anxiety and sleep problems in youth and socioeconomic status.^74,75^ Finally, while the study provides insights into user acceptability of the SMILE app, only an efficacy trial will test its ability to alleviate the burden on youth with anxiety and sleep problems.

## Conclusions

In conclusion, this qualitative study marks an important stride in addressing the mental health needs of youth dealing with co-morbid anxiety and sleep problems. The positive reception of the SMILE app, coupled with constructive feedback, positions it to be refined into an impactful, user-friendly intervention. The study contributes to the growing body of evidence supporting mental health apps as accessible, helpful, and timely interventions, particularly when aligned with the preferences and needs of youth users. By integrating mobile sensing technology to extend tracking and personalization abilities, the SMILE app has the potential to modernize the delivery of mental health care. The recommendations and suggestions we collected are indispensable in ensuring that future iterations of the SMILE app have a meaningful impact on the mental health of youth facing anxiety and sleep problems. Ongoing research and development will be imperative to fully realize the SMILE app's potential and ensure its meaningful impact on the mental well-being of youth.

## Supplemental Material

sj-docx-1-dhj-10.1177_20552076241283242 - Supplemental material for Sleep well, worry less: A co-design study for the development of the SMILE app Supplemental material, sj-docx-1-dhj-10.1177_20552076241283242 for Sleep well, worry less: A co-design study for the development of the SMILE app by Marcus Cormier, Matt Orr, Alanna Kaser, Hannah MacDonald, Jill Chorney and Sandra Meier in DIGITAL HEALTH

sj-pdf-2-dhj-10.1177_20552076241283242 - Supplemental material for Sleep well, worry less: A co-design study for the development of the SMILE app Supplemental material, sj-pdf-2-dhj-10.1177_20552076241283242 for Sleep well, worry less: A co-design study for the development of the SMILE app by Marcus Cormier, Matt Orr, Alanna Kaser, Hannah MacDonald, Jill Chorney and Sandra Meier in DIGITAL HEALTH
