# Supplementary material for: Sleep well, worry less: A co-design study for the development of the SMILE app
Source: Digit Health. 2024 Sep 25;10:20552076241283242. doi: 10.1177/20552076241283242 (PMC11468482; doi:10.1177/20552076241283242)
Supplement: sj-docx-1-dhj-10.1177_20552076241283242 - Supplemental material for Sleep well, worry less: A co-design study for the development of the SMILE app [file sj-docx-1-dhj-10.1177_20552076241283242.docx]

**Screening Questionnaire**

The following questions ask for some information about you to determine if you are eligible for participating in the co-design study for the SMILE app. This questionnaire will take approximately 5 minutes to complete.

What is your age in years? __________________________________

Do you experience anxiety?

- Yes
- No

Do you experience sleep problems (e.g., trouble getting to sleep, trouble staying asleep, waking up through the night)?

- Yes
- No

Can you read English?

- Yes
- No

Do you speak English?

- Yes
- No

Do you currently own a smartphone?

- Yes
- No

Are you open to attend a session (online or in-person options) to view and provide feedback on the SMILE app prototype?

- Yes
- No

*(If ineligible age or responded “No” to any items):*

Thank you for your interest in participating in the study! Based on your responses to the above questions, you are not eligible to participate. As such, you will receive no further communication about this study. Please click the "Submit" button below to finish this survey.

*(If eligible age and responded “Yes” to all item):*

Thank you for your interest in participating in the study! Based on your responses to the above questions, you are eligible to participate. Please click the "Next" button to proceed to the Information and Consent Form, which will provide more details about what participation will involve.

**Consent Information**

**Development of Sleep Intervention Features for a Smartphone Intervention for Anxiety in Youths**

**Study Contact Information:**

SMILE Study

C/O Dr. Matthew Orr

Halifax, Nova Scotia | B3K 6R8

Email: SMILE@iwk.nshealth.ca

Toll Free: 1-833-905-0342

**Study Primary Investigator:**

Dr. Matthew Orr, Postdoctoral Researcher Department of Psychiatry, Dalhousie University

Phone: 902-452-6702 E-mail: matt.orr@dal.ca

**Supervising Investigator:**

Dr. Sandra Meier, Associate Professor Faculty of Medicine, Dalhousie University, IWK Health Centre

Phone: 902-470-7720 E-mail: Sandra.Meier@iwk.nshealth.ca

**Study Co-Investigators:**

Silvia Marin-Dragu, Dalhousie University, Halifax, NS

**Introduction:**

We are asking you to take part in a study. We want to hear from youths (15-25 years) who have sleep problems and anxiety problems. Specifically, we want to learn about your experiences with mobile apps and your perspective on a new mobile app for anxiety and sleep problems. The study information will be used to help us understand how to design an app to help youths with anxiety and sleep problems. We believe that mental health apps might be helpful as youths could use them wherever they are and whenever they like. We hope that findings from the study will help to improve youths’ anxiety and sleep problems. Please read the information about this study below. This form provides information about the study, such as the purpose of the study, the risks and benefits, and what you will be asked to do. Before you decide if you want to take part, it is important that you understand the study details. A staff member from our research team is available to answer any questions you have about the study at our toll-free number 1-833-905-0342 or by email at SMILE@iwk.nshealth.ca. You do not have to take part in this study; taking part is entirely voluntary (your choice).

**Why are the researchers doing the study?**

We would like to learn about your experiences as a youth with anxiety and sleep problems and your perceptions of a new app. We want to help you with your anxiety and sleep problems. We have designed a mobile app to improve anxiety and sleep problems in youth. To make sure this app is helpful to youths, we are asking for your perspectives on the app.

**Where will this study take place?**

This study is taking place at the IWK Health Centre in Halifax, NS.

**What will I be asked to do?**

We hope to include a total of 12 youths in this study. You will complete three online questionnaires about you, your anxiety problems, and your sleep problems. It will take about 35 minutes to complete all of the questionnaires. Next, you will take part in a workshop, during which we will ask about your thoughts about the mobile app and how we could improve it. Workshops will take approximately 60 minutes to complete. Workshops will be conducted in two ways: (1) at the IWK Health Centre and (2) online via Zoom. For both methods, will be audio and video recorded. We will save what you are saying to text and delete the audio sample afterwards. Video will be used to record the emotions expressed through facial expression (e.g., happiness, sadness, anger) and the video files will be deleted afterwards. Access to your medical charts is not necessary.

**What are the burdens, harms, and potential risks to taking part in this study?**

Some questions on the online questionnaires may remind you of sad events. You do NOT have to answer any questions that make you feel uneasy. You may contact our study team any time if you would like to talk about your feelings. Contact information is at the top of this page. If we learn that you are in danger of harming yourself or others, you will be asked to seek help. There is no cost to you for taking part in the study, but you will need to be able to attend the workshops in person at the IWK Health Centre.

**What are the possible benefits to taking part in this study?**

There may be no direct benefit to you or your family. The information from this study will be used to help youths with anxiety and sleep problems.

You will also receive a $20 reimbursement in the form of an Amazon gift card.

**How will my privacy be protected?**

Everything you do in this study will be kept private and confidential. All information you give during the workshop is confidential. All data will be stored on secure servers at the IWK Health Centre.

Your data will be used for writing scientific articles and reports. Workshop data will be analyzed and reported in an aggregated form, which means none of your data will be identifiable and absolutely none of your personal information will be shared.

**How will my data be stored?**

All personal information you provide in the online questionnaires will be treated as private and confidential. The data will be stored on a secure server. All identifying information, such as name and contact information, will be replaced with study ID numbers. Your name and contact information will be excluded from databases and reports. Five years after the study, all private information (like your name) will be permanently removed.

The IWK Research Ethics Board, or other funders of this study could review study information (audit the study) to make sure that the research is being done properly. In the case of an audit, your file, which may include your name and information that you have provided during the study, could be reviewed.

**Can I withdraw or stop taking part from the study?**

Yes, you will be able to withdraw at any timepoint. However, if you inform our study team more than 1 month after you completed the interview that you would like to withdraw, we may have already used your data in our analysis and would not be able to withdraw these responses.

**What are my research rights?**

Completing this Information and Consent form indicates that you have agreed to take part in this research and for your response data to be used. In no way does this waive your legal rights nor release the investigator(s), sponsors, or involved institutions(s) from their legal and professional responsibilities. Choosing not to take part will in no way affect the care you and/or your child currently receives or will receive in the future. If you become ill or injured as a direct result of participating in this study, necessary medical treatment will be available at no additional cost to you. You are free to withdraw from the study at any time without jeopardizing the health care you are entitled to receive. If you have any questions at any time during or after the study about research in general you may contact the Research Office of the IWK Health Centre at 902-470-8717, Monday to Friday between 8:00am and 4:00pm. If at any point throughout the focus group you are feeling that you may be in immediate danger of seriously harming yourself or someone else, you should seek help right away by going to your nearest emergency room or calling 911. You may also call Crisis Services Canada, at 1-833-456-4566.

Please answer the following questions prior to providing consent:

Do you understand that interviews will be used in a research study asking about a mobile health app for youths?

- Yes
- No

Were you able to ask questions and discuss this study?

- Yes
- No

Do you understand who will see your data?

- Yes
- No

Do you understand that you can stop participating at any time?

- Yes
- No

Do you agree to be in this study?

- Yes
- No

Would you like to be contacted for future studies (e.g., other online surveys, focus group or treatment projects)?

- Yes
- No

Would you like to receive the results of this study?

- Yes
- No

Please enter your name: __________________________________

Please enter your email address: __________________________________

Today's date: __________________________________

Thank you for taking the time to review the Information and Consent Form for the study Development of Sleep Intervention Features for a Smartphone Intervention for Anxiety in Youths and for consenting to participate in the study! Please press the “submit” button to continue to the Participant Characteristics Questionnaire.

**Demographic Questionnaire**

Instructions: The following questions ask for some basic information about you. This will allow the research team to describe, as a group, the study sample. This questionnaire will take approximately 5 minutes to complete.

Your age

- 15
- 16
- 17
- 18
- 19
- 20
- 21
- 22
- 23
- 24
- 25

Your gender

- Man
- Woman
- Other, please specify:

Other:

__________________________________

Your sex

- Male
- Female
- Other, please specify:

Other: __________________________________

How would you best describe your ethnic or cultural heritage?

- White
- Black
- Aboriginal
- South Asian
- Chinese
- Filipino
- Latin-American
- Arab
- West Asian
- South East Asian
- Korean
- Japanese
- Other, please specify

Other: __________________________________

How would you describe the community where you live?

- Rural
- Town
- City under 500,000 people
- City over 500,000 people

**SCAARED**

Instructions: Below is a list of sentences that describe how people feel. Read each phrase and decide if it is “Not True or Hardly Ever True” or “Somewhat True or Sometimes True” or “Very True or Often True” for you. Then for each sentence, fill in one circle that corresponds to the response that seems to describe you for the last 3 months.

|  | Not True or Hardly Ever True | Somewhat True or Sometimes True | Very True or Often True |
| --- | --- | --- | --- |
| 1) When I feel nervous, it is hard for me to breathe | O | O | O |
| 2) I get headaches when I am at school, at work or in public places | O | O | O |
| 3) I don't like to be with people I don't know well | O | O | O |
| 4) I get nervous if I sleep away from home | O | O | O |
| 5) I worry about people liking me | O | O | O |
| 6) When I get anxious, I feel like passing out | O | O | O |
| 7) I am nervous | O | O | O |
| 8) It is hard for me to stop worrying | O | O | O |
| 9) People tell me that I look nervous | O | O | O |
| 10) I feel nervous with people I don't know well | O | O | O |
| 11) I get stomach aches at school, at work, or in public places | O | O | O |
| 12) When I get anxious, I feel like I'm going crazy | O | O | O |
| 13) I worry about sleeping alone | O | O | O |
| 14) I worry about being as good as other people | O | O | O |
| 15) When I get anxious, I feel like things are not real | O | O | O |
| 16) I have nightmares about something bad happening to my family | O | O | O |
| 17) I worry about going to work or school, or to public places | O | O | O |
| 18) When I get anxious, my heart beats fast | O | O | O |
| 19) I get shaky | O | O | O |
| 20) I have nightmares about something bad happening to me | O | O | O |
| 21) I worry about things working out for me | O | O | O |
| 22) When I get anxious, I sweat a lot | O | O | O |
| 23) I am a worrier | O | O | O |
| 24) When I worry a lot, I have trouble sleeping | O | O | O |
| 25) I get really frightened for no reason at all | O | O | O |
| 26) I am afraid to be alone in the house | O | O | O |
| 27) It is hard for me to talk with people I don't know well | O | O | O |
| 28) When I get anxious, I feel like I'm choking | O | O | O |
| 29) People tell me that I worry too much | O | O | O |
| 30) I don't like to be away from my family | O | O | O |
| 31) When I worry a lot, I feel restless | O | O | O |
| 32) I am afraid of having anxiety (or panic) attacks | O | O | O |
| 33) I worry that something bad might happen to my family | O | O | O |
| 34) I feel shy with people I don't know well | O | O | O |
| 35) I worry about what is going to happen in the future | O | O | O |
| 36) When I get anxious, I feel like throwing up | O | O | O |
| 37) I worry about how well I do things | O | O | O |
| 38) I am afraid to go outside or to crowded places by myself | O | O | O |
| 39) I worry about things that have already happened | O | O | O |
| 40) When I get anxious, I feel dizzy | O | O | O |
| 41) I feel nervous when I am with other people and I have to do something while they watch me (for example: speak, play a sport.) | O | O | O |
| 42) I feel nervous when I go to parties, dances, or any place where there will be people that I don't know well | O | O | O |
| 43) I am shy | O | O | O |
| 43) When I worry a lot, I feel irritable | O | O | O |

**SCARED**

Instructions: Below is a list of sentences that describe how people feel. Read each phrase and decide if it is “Not True or Hardly Ever True” or “Somewhat True or Sometimes True” or “Very True or Often True” for you. Then for each sentence, fill in one circle that corresponds to the response that seems to describe you for the last 3 months.

|  | Not True or Hardly Ever True | Somewhat True or Sometimes True | Very True or Often True |
| --- | --- | --- | --- |
| 1) When I feel frightened, it is hard for me to breathe | O | O | O |
| 2) I get headaches when I am at school | O | O | O |
| 3) I don't like to be with people I don't know well | O | O | O |
| 4) I get nervous if I sleep away from home | O | O | O |
| 5) I worry about people liking me | O | O | O |
| 6) When I get frightened, I feel like passing out | O | O | O |
| 7) I am nervous | O | O | O |
| 8) I follow my mother or father wherever they go | O | O | O |
| 9) People tell me that I look nervous | O | O | O |
| 10) I feel nervous with people I don't know well | O | O | O |
| 11) I get stomach aches at school | O | O | O |
| 12) When I get frightened, I feel like I'm going crazy | O | O | O |
| 13) I worry about sleeping alone | O | O | O |
| 14) I worry about being as good as other kids | O | O | O |
| 15) When I get frightened, I feel like things are not real | O | O | O |
| 16) I have nightmares about something bad happening to my parents | O | O | O |
| 17) I worry about going to school | O | O | O |
| 18) When I get frightened, my heart beats fast | O | O | O |
| 19) I get shaky | O | O | O |
| 20) I have nightmares about something bad happening to me | O | O | O |
| 21) I worry about things working out for me | O | O | O |
| 22) When I get frightened, I sweat a lot | O | O | O |
| 23) I am a worrier | O | O | O |
| 24) When I worry a lot, I have trouble sleeping | O | O | O |
| 25) I am afraid to be alone in the house | O | O | O |
| 26) It is hard for me to talk with people I don't know well | O | O | O |
| 27) When I get frightened, I feel like I'm choking | O | O | O |
| 28) People tell me that I worry too much | O | O | O |
| 29) I don't like to be away from my family | O | O | O |
| 30) I am afraid of having anxiety (or panic) attacks | O | O | O |
| 31) I worry that something bad might happen to my parents | O | O | O |
| 32) I feel shy with people I don't know well | O | O | O |
| 33) I worry about what is going to happen in the future | O | O | O |
| 34) When I get frightened, I feel like throwing up | O | O | O |
| 35) I worry about how well I do things | O | O | O |
| 36) I am scared to go to school | O | O | O |
| 37) I worry about things that have already happened | O | O | O |
| 38) When I get frightened, I feel dizzy | O | O | O |
| 39) I feel nervous when I am with other children or adults and I have to do something while they watch me (for example: read aloud, speak, play a game, play a sport.) | O | O | O |
| 40) I feel nervous when I go to parties, dances, or any place where there will be people that I don't know well | O | O | O |
| 41) I am shy | O | O | O |

**Insomnia Severity Index**

Instructions: For each question, please select the number that best describes your answer.

**Please rate the CURRENT (i.e., LAST 2 WEEKS) SEVERITY of your insomnia problem(s).**

|  | None | Mild | Moderate | Severe | Very Severe |
| --- | --- | --- | --- | --- | --- |
| 1) Difficulty falling asleep | O | O | O | O | O |
| 2) Difficulty staying asleep | O | O | O | O | O |
| 3) Problems waking up too early | O | O | O | O | O |

4) How SATISFIED/DISSATISFIED are you with your CURRENT sleep pattern?

- Very Satisfied - 0
- Satisfied - 1
- Moderately Satisfied - 2
- Dissatisfied - 3
- Very Dissatisfied - 4

5) How NOTICEABLE to others do you think your sleep problem is in terms of impairing the quality of your life?

- Not at all noticeable - 0
- Barely - 1
- Somewhat - 2
- Much noticeable - 3
- Very noticeable - 4

6) How WORRIED/DISTRESSED are you about your current sleep problem?

- Not at all - 0
- A little - 1
- Somewhat - 2
- Much - 3
- Very much - 4

7) To what extent do you consider your sleep problem to INTERFERE with your daily functioning (e.g. daytime fatigue, mood, ability to function at work/daily chores, concentration, memory, mood, etc.) CURRENTLY?

- Not at all interfering - 0
- A little - 1
- Somewhat - 2
- Much - 3
- Very much interfering - 4
